# Prevalence of Caries and Associated Risk Factors in a Representative Group of Preschool Children from an Urban Area with High Income in Milan Province, Italy

**DOI:** 10.3390/ijerph17103372

**Published:** 2020-05-12

**Authors:** Alessandro Nota, Atanaz Darvizeh, Jasmina Primožič, Federica Onida, Floriana Bosco, Enrico Felice Gherlone, Simona Tecco

**Affiliations:** 1 Dental School, Vita-Salute San Raffaele University and IRCCS San Raffaele Hospital, 20132 Milan, Italy; a.nota@docenti.unisr.it (A.N.); atanazdarvizeh@gmail.com (A.D.); fede686@hotmail.it (F.O.); florianabosco@gmail.com (F.B.); gherlone.enrico@hsr.it (E.F.G.); 2 Department of Orthodontics, University of Ljubljana, 1000 Ljubljana, Slovenia; jasminaprimozic@gmail.com

**Keywords:** caries, prevalence, preschool children, high income population, urban population

## Abstract

The present survey provides a quantitative assessment of caries prevalence, covering a group of 3–5 year-old children from an urban area generally with a high income in the province of Milan, and a comparison of the obtained results with the data presented by the W.H.O. A cross-sectional study was conducted in the period from March to September 2018 to investigate the prevalence of caries in a sample of 160 children (82 females and 78 males). The absence/presence of caries was defined as a dependent variable. Factors concerning lifestyle, diet, oral habits, oral hygiene, the presence and type of malocclusion and mouth breathing attitude were considered as risk factors. Data were analyzed by Chi-square (χ2) and regression tests using SPSS (version 25.0) software. In total, 84.38% of children (135 out of 160) showed no caries. A regression analysis demonstrated that children who had already received an early first dental visit were mostly those already affected by caries. Furthermore, children who had four meals daily or more were less exposed to the risk of developing caries compared to those who had only 1–3 meals daily. The caries prevalence of preschool children from urban areas with a high income in Milan province is relatively close to that considered acceptable by the W.H.O. in its proposed goals for the year 2020. Therefore, it can be concluded from the obtained results that there is a possibility for further improvement in preventing caries growth at its initial stage: it is necessary for the number of meals daily consumed by children to be controlled by parents, and conducting a dental visit early in childhood must not be neglected.

## 1. Introduction

Dental caries is one of the most widespread childhood oral diseases in the world [1]. Once it occurs, its manifestation persists throughout life, even after it has been treated, possibly due to an experience with pain and anxiety associated to dental fear [2]. Thus, primary prevention is necessary in the early phase of childhood to reduce the risk of caries initiation and to avoid its further development. The preschool period is the time in which deleterious oral habits, caries patterns and risk factors are established. Consequently, it is also the ideal period to intervene and create healthy habits in order to establish a permanent protective influence, as performed for other conditions such as poor posture and malocclusions [3].

The W.H.O. project for the year 2020 states that the number of children aged 3 to 5 years with coronal caries or the presence of filled teeth in at least one primary tooth should be decreased to 10% of subjects (see https://www.healthypeople.gov/node/4992/data_details). It has been presented in published research works that there may be a relationship between lifestyles and caries risk in various social groups; for example, low or high-income families [4] as well as urban or rural households [5,6]. In particular, family income status and its variability over time have also been proven to affect the treatment patterns and frequency of dental caries among children [4]; this was also recently observed among Italian children aged between 5 and 12 [7]. It is well known that the lifestyle of a target population is directly influenced by the geographical area in which the subjects live [6]. For these reasons, it is essential that the characteristics of a targeted population should be well defined in order to address the proper preventive programs and to put suitable strategies into action [6]. Thus, updating the information related to caries and the specific risk factors among preschool children should always be taken into consideration, keeping in mind the geographical area of interest and the population from which the data was drawn, for optimized community planning. In the last decade, only a few epidemiological studies have focused on caries prevalence among preschool children in Europe. To the best of the authors’ knowledge, the most recent epidemiological studies in this field use data reported in 2005 in the Netherlands related to a representative group of 5 year-old children [8], data presented in 2007 in North East London boroughs regarding children aged 3–4 years [9], data published in 2007–2008 in Scotland with a sample of 5 years old children [5], and more recently, data documented in 2013–2014 on 3–6 year-old children in Poland [10]. From these surveys, the prevalence of caries in European preschool children appeared to remain to be too high, as it ranged from 18.21% to 66.04%, which was far from the W.H.O. objectives for 2020; this suggests that, despite the common belief concerning the importance of oral disease prevention, the general lifestyles of European families are far from being ideal. Thus, it seems appropriate to collect data regarding the prevalence of caries, defining the current situation of the different socio-cultural groups living in Europe, and designing further preventive programs which may lead to the 2020 W.H.O. objectives being met [11].

Against this background, the present survey aims to begin an oral health and preventive dentistry project directed to a population of 3–5 year-old children from an urban area with a generally high income in the province of Milan. Therefore, the aims of the present study were to collect data and calculate the prevalence of caries in this representative group, comparing the results with the objectives proposed by W.H.O. Furthermore, we aimed to analyze the correlation of caries with the potential risk factors related to the studied children’s lifestyle and individuate the strategies to improve primary prevention projects. The considered potential risk factors are related to lifestyles, eating habits, and oral hygiene [12]; in addition, we considered the presence and type of malocclusions, non-nutritive sucking habits and mouth breathing attitudes [13,14,15,16].

## 2. Materials and Methods 

A cross-sectional study was conducted in the period from March to September 2018 to investigate the prevalence of caries in a sample of preschool children aged 3–5 years from an urban area with high income in Milan province, Italy. The main objective was to investigate the association of dental caries with risk factors such as lifestyles, eating habits, oral hygiene, presence and type of malocclusions, non-nutritive sucking habits and mouth breathing. A series of visits and meetings were carried out in the children’s classrooms at the “Fiume” kindergarten (Vimodrone, Milan, Italy) by two dentists and a student attending the last year of a dental hygiene course. The total number of children included in the study was 160 (82 females and 78 males). The town of Vimodrone is a municipality that is part of the geographical area known as the “the greater area of Milan”, the fourth largest metropolitan area of Europe in terms of GDP (Gross Domestic Product), and the sixth in terms of pro-capita GDP (data from Eurostat 2011–2013), following capital cities such as Paris (623 billion euros of GDP), London (617 billion euros) and Madrid (332 billion euros). The GDP of “the greater area of Milan” reached almost 185 billion euros; thus, the region is highly representative of a high-income urban area in Europe. The present protocol was approved by the Ethical Committee of the Vita-Salute San Raffaele University (Milan, Italy) (Document V8 of the 2-7-2015 of the Ethic Committee of the San Raffaele Hospital). Informed consent was obtained from parents/caregivers prior to the distribution of questionnaires and to the oral health examination. 

The present project was organized in the following way. Firstly, a questionnaire about lifestyles, eating habits and oral hygiene was prepared by a group of expert dentists on the basis of potential variables observed after a summary of the previous literature [16,17,18,19,20,21]. The quantitative content validity of the questionnaire was obtained using Waltz and Bausell’s method. In order to achieve validity, a group of dentists was provided with a Content Validity Index form (CVI) to identify the relevance, clarity and simplicity of each question based on four-part Likert scale. The CVI formula was utilized to calculate the grade of each question individually. All the questions with a value less than 0.70 were considered unacceptable and deleted. The reliability of the questionnaire was established with the aid of the split half method in order to assure its internal consistency. The final score of the reliability coefficient, which was measured using the Spearman–Brown formula, presented a value close to one, which confirmed the reliability of the questionnaire. After the final version of the questionnaire was prepared, a meeting with the children was scheduled at school, during which a game-model lesson appropriate for the age of the children was carried out by the doctors, explaining the basic rules regarding a correct diet and habits of oral hygiene at home. Then, the validated questionnaire was given to the children’s Primary Caregivers (PCG), who were asked to complete it honestly and with great attention before the next meeting. 

At the following appointment, an intra-oral clinical examination of the children was performed in order to detect the presence/absence of caries, the number of caries and filled teeth, the caries position on each tooth, the presence and type of malocclusion and the breathing habit. The examination was conducted according to sanitary requirements using adequate artificial lighting, nitrile gloves, a sterile disposable probe and mirror. Dental examination results were registered on an appropriate diagram. 

Clinical data of caries were considered in relation with the other variables to verify whether or not there was a correlation between them. The frequencies for each variable were calculated. The absence/presence of caries was considered as the dependent variable to be potentially associated with the variables concerning the breastfeeding effect (S1 and S2), the use of pacifiers (S3 and S4), non-nutritive oral habits (S5, S6 and S7), the breathing habit (S8), early dental visits (S9, S10 and S11), lifestyle/sport activities (S13 and S14), eating habits (from S15 to S25), oral hygiene habits (S26, S27 and S28), clinical data about filled teeth (S31 and S32), and the clinical data about malocclusions (S33, S34 and S12). To further illuminate any potential role of lifestyle/eating habits, for the number of meals consumed daily (S15), the habit to have breakfast at home (S16), and the control of eating habits by parents (S25), separate analyses were performed. Associations were analyzed using χ2 test. Then, the variables which showed a statistically significant association were introduced to a multivariate logistic regression model in order to evaluate their influence with the presence/absence of caries considered as the dependent variable. Data were analyzed using SPSS 25.0 software (IBM Corp. Released 2011. IBM SPSS Statistics for Windows, Version 25.0. Armonk, NY, USA: IBM Corp). The significance threshold was set at 0.05.

## 3. Results

The total sample taken into consideration in this cross-sectional study included 160 children aged between 3 and 5 years, with a gender distribution of 78 males (48.75%) and 82 females (51.25%). Table 1 describes the demographic characteristics of the present sample.

In total, 135 children out of 160 (84.38%) had no caries, 20 children out of 160 (12.5%) had up to three caries, and five children out of 160 (3.13%) had more than four caries.

Table 2 describes the results deduced from the questionnaire as frequencies and percentages identifying the statistical significance after implementing the χ2 test. The bad oral habits, presence/absence of breastfeeding, thumb sucking, night oral breathing and pacifier usage duration were found to be statistically significant associated variables. Furthermore, the results indicate that lifestyle, attendance to the first dental visit, frequency of sport practice and number of meals per day were significantly associated variables. In addition, the number of teeth brushing sessions per day as well as the number of restorations and the presence/absence of malocclusion were recognized as significantly associated oral variables.

Table 3 reports the results of the multivariate logistic regressions. Children who had already received an early first dental visit (previous to the beginning of the project) were more often those already affected by caries. 

Table 4 reports the regression between caries and the number of meals per day, demonstrating that children who had four meals daily, or more, were less exposed to the risk of developing caries compared to those who had only 1–3 per day. 

## 4. Discussion

This observational study reports data affecting the prevalence of dental caries in children aged 3–5 years, from a high-income community living in an urban area in Milan province, Italy, which is representative of a high-income community in Southern Europe. To the best of the authors’ knowledge, this is a unique report concerning pre-school children from high-income families of this geographical area. Thus, it seems that the present data may have the potential to be generalized for application in any similar geographical area and social characteristics, at least in Southern Europe. 

The aim of this work was to evaluate how the quantitative occurrence of caries can be influenced by potentially associated causes. The prevalence of caries was compared with the goal set by W.H.O. for the year 2020. The present survey indicates that 15.6% of 160 children showed almost one decayed/filled tooth. It is clear that the objective proposed by W.H.O. has not yet been achieved; therefore, additional preventive measures seem to be necessary and should be implemented. The prevalence observed in the present study is remarkably higher than the value of x8.2% reported by Colombo et al. [22] for early childhood caries (ECC) (age < 71 months), but it is just slightly higher than the 14.7% reported by the same authors for children aged 4–6 years concerning the whole Italian population, where a higher prevalence should be expected [23]. This shows a probable underestimation of the real amount of ECC; Colombo et al. noted the use of a parent self-report questionnaire for the presence of caries as a limitation of their study, which could affect the validity of their results. However, in the present study, the caries diagnosis was performed by professionals and this eliminates this inaccuracy, leading to a higher degree of validity. Furthermore, the present data can be compared with the results from the previous surveys recorded in Europe to evaluate the criticality of the problem in this territory. 

Similar to the present data are those extracted in 2007 from a survey from North East London boroughs on children aged 3–4 years, among which a percentage of 18.21% (26 out of 70 whose parents were white European) showed at least one caries [9]. The data of these surveys suggest a comparative trend of caries prevalence between these two industrialized areas of Europe.

From the results of a survey carried out in the Netherlands and published in 2005, a meaningful difference from the present data can be observed. The sample used in their study consisted of 386 children aged 5 years, representing those with a lower socioeconomic status; a percentage of 56% of children (171 out of 386) showed at least one decayed/filled tooth [8]. The main source of difference seems to be the different economic status of the families, and to the fact that Dutch children were all aged 5 years. In fact, Dutch children from higher social classes were reported to have a lower percentage of caries on average compared to those from lower classes, but the report into this was dated before 2000, and for this reason, it is not directly comparable with the present data [24]. 

Another database recorded in 2007–2008 in Scotland with 5 year-old children from “accessible towns” showed 42.3% prevalence of at least one caries, which is significantly higher than our result. This difference in the Scottish sample could be due to the fact that Levin et al. considered only children aged 5 years and counted also deciduous teeth lost due to caries based on a national oral health survey; in addition, they did not differentiate high/low-income areas [10]. 

Finally, Kamińsk et al. (2013–2014) presented a group of 3–6 year-old children (636 subjects) from an urban area of Poland with the frequency of caries ranging from 40.91% to 66.04%; however, their results are not fully comparable to ours due to the fact that high and low-income groups were not distinguished in their study [11]. 

The summarized comparison with other surveys from Europe confirms that the present data is the first recent report on caries prevalence in a representative sample including a high-income urban area of Southern Europe. It also indicates that the present clinical situation is encouraging, and children from the present sample can be considered to be in a good state of oral health with a promising trend that seems to be able to become proximal to the aims of the W.H.O. for 2020. For this reason, preventive measures should be implemented throughout this territory to completely achieve the goal set by the W.H.O. for 2020. 

From the present data, it can be observed that factors such as oral hygiene routine, breastfeeding, non-nutritive sucking habit, breathing pattern, use of a pacifier, lifestyle/sport activities, clinical data about oral health and malocclusions do not generally affect the prevalence of caries among preschool children. This can suggest that the presence of caries at this age could be more related to other predisposing factors (e.g., genetics) rather than incorrect behaviors; with this in mind, the children were very young, and poor oral habits could not affect their oral health status yet. However, the multiple regression analysis showed a statistically significant association between the presence of caries and a previous dental examination prior to the beginning of this project (Table 3). This result suggests that the percentage of PCG (only 27.41%) who brought their child to the dentist for a preventive check-up visit (without urgent problems such as toothache) remains too low. This behavior is probably connected to the level of oral health knowledge by the PCG, which is very often related to the first dental visit for a dental problem such as pain or the beginning of primary teeth exfoliation. This observation agrees with a recent survey from Saudi Arabia, which states that most parents bring their children to a dentist only after experiencing pain or suffering from dental caries. About 37.33% (in a sample of 320 PCG from Saudi Arabia) of participants said that pain was the main reason for them to take their children to visit a dentist [19]. Information campaigns regarding oral care should encourage a first preventive dental visit in children from 3 to 5 years. This early preventive visit should be mainly focused on providing the PCG with necessary information regarding preventive practices to avoid the onset of problems for them [25], and for their children, rather than planning only for the intra-oral examination of children.

In addition, the present survey reveals a significant association between the presence of caries and the number of daily meals consumed (Table 4). This result is in contrast with the generally held belief that a high frequency of food intake leads to a lowering of intra-oral pH, causing a high risk of developing caries. On the contrary, the present data reveal that a high frequency of daily meals does not correspond to an equally high level of caries. It could be hypothesized that the higher number of meals increases oral hygiene operations for caries control. Therefore, it is necessary to ascertain which types of food had been consumed by the children, as the type of food can influence the formation of caries (cariogenic and non-cariogenic foods). The present survey confirmed that the majority of the participating children consumed a Mediterranean-style diet [20] and failed to evince a correlation between eating habits and the occurrence of caries; however, it would be interesting to conduct a more comprehensive study concerning eating style with a larger sample to monitor the type of food consumed during the day. Nevertheless, another recent study showed the importance of parental control on children’s eating behavior and its impact on the development of dental caries [26].

In summary, the observed findings suggest the importance of projects being carried out in nursery schools, where dental professionals and dental hygienists can organize meetings with the PCG to raise awareness on early dental visits and eating habits. These meetings would aim to inform parents about the prevention practices to be implemented to change the risky behaviors of children, preventing the onset of diseases in the oral cavity. In particular, from the present survey, it seems that oral hygiene procedures appear to be well known by PCGs, but they lack sufficient information regarding the crucial role of a preventive approach, such as early dental visits. It is essential also to convince PCGs to make the first dental visit to gain advice concerning preventive approaches to caries diseases, or for traumatic injuries to teeth or the temporomandibular joint [27], or for malocclusions, and not only to visit the dentist after the occurrence of a painful experience. A recent study from Croatia [21] confirmed that the ignorance of PCGs about oral health leads to an irresponsible behavior, and this may be the main source of undesirable poor oral health conditions in that geographical area. The present findings confirm that the caries status of preschool children from industrialized areas such as Milan in southern Europe is mainly affected by the education level of the PCG; by elevating their awareness, a substantial contribution can be made to altering their oral health attitude and consequently improving the oral health of their children [28].

The limitations of this study which may have influenced the results were the size of the target population and the number of children; to achieve more generalizable results, the sample could be enlarged by including more schools. The second limitation the fact that the intra-oral examinations were conducted at school, which was influenced by the lack of technical equipment and the position of the child, as this can restrict the vision of the operator.

## 5. Conclusions

The results of the present study state that caries prevalence among preschool children from urban areas in Milan with a high income seems acceptable and to exhibit a promising trend; however, the results continue not to meet the aims of the W.H.O. for the year 2020. It can be concluded from the obtained results that there is a possibility for further improvement by the prevention of caries growth in its initial stage: the number of meals daily consumed by children needs to be controlled by parents, and an early childhood dental visit must not be neglected. Further prospective studies are needed to validate the findings of the present study.

## Figures and Tables

**Table 1:** Demographic data (age and gender) of the investigated sample.

Age (Years)	Number of Children in the Whole Sample	Males	Females
3	45	22	23
4	49	21	28
5	66	35	31

**Table 2:** Frequencies, percentages, and Chi-square values for each point of the questionnaire.

Topic	Code	Question	Answer	Frequency	%	Chi-Square (*p*)
Breastfeeding effect	S1	Feeding	Breastfeeding	99	61.9	167.225 (*p* = 0.000)
			Artificial	43	26.9	
			Mixed	18	11.2	
	S2	For how many months?	2-14 month	121	75.6	
			15-26 month	28	17.5	
			27-38 month	11	6.9	
Pacifier usage	S3	For how many months did your child use the pacifier?	3-22 month	86	53.8	47.000 (*p* = 0.000)
			23-41 month	70	43.8	
			42-60 month	4	2.5	
	S4	When did your child used the pacifier?	Just to fall asleep	71	44.4	
			Only when he cried	26	16.2	
			Always	63	39.4	
Non-nutritive oral habits	S5	Does your child have finger sucking habit?	No	150	93.8	408.600 (*p* = 0.000)
			Yes	10	6.2	
	S6	If so, when?	Just to fall asleep	3	1.9	
			Just to relax	5	3.1	
			Always	2	1.2	
			Doesn’t have	150	93.8	
	S7	The child has the habit of:	Lip sucking/cheek sucking	7	4.4	
			Nail biting	6	3.8	
			Put pens or pencils in the mouth	6	3.8	
			Grinding teeth	16	10.0	
			Other	18	11.2	
			No oral habit	107	66.9	
Breathing habits	S8	How does the child breathe while sleeping?	Mouth	40	25.0	40.000 (*p* = 0.000)
			Nose	120	75.0	
Dental visits	S9	Has the child ever had a dental visit?	No	107	66.9	175.375 (*p* = 0.000)
			Yes	53	33.1	
	S10	If so, at what age?	<3 years	18	11.2	
			3-5 years	35	21.9	
			Never	107	66.9	
	S11	What was the reason for the dental visit?	Pain	3	1.9	
			Caries	20	12.5	
			Control	28	17.5	
			Orthodontics	2	1.2	
			Never	107	66.9	
Clinical data on malocclusion	S12	Does your child wear an orthodontic appliance?	No	159	99.4	
			Yes	1	0.6	
Lifestyle/Sport activities	S13	How many times does your child play sport?	Never	52	32.5	22.775 (*p* = 0.000)
			Sometime	9	5.6	
			2-3 times per week	52	32.5	
			Everyday	47	29.4	
	S14	Which sport does your child prefer?	Swimming	70	43.8	
			Other	50	31.2	
			No sport	40	25.0	
Eating habits	S15 *	How many meals does your child eat per day?	1-3 meals	50	31.2	58.075 (*p* = 0.000)
		* Chi-square 22.500(*p* = 0.000)	>3	110	68.8	
	S16 *	Does your child eat breakfast at home?	Sometime	18	11.2	
		* Chi-square 96.100(*p* = 0.000)	Always	142	88.8	
	S17	What does your child usually eat for breakfast?	Dairy	31	19.4	
			Grains	30	18.8	
			Sweets	23	14.4	
			Pizzette	19	11.9	
			Egg	20	12.5	
			Tea, juice	33	20.6	
			Mix of all	4	2.5	
	S18	Does your child snack during morning/afternoon?	No	4	2.5	
			Sometime	21	13.1	
			Yes	135	84.4	
	S19	Type of snack?	Carbs	32	20.0	
			Yoghurt, fruit	33	20.6	
			Sweet snacks	35	21.9	
			Sugary drink	20	12.5	
			Snack given by the school	37	23.1	
			Others	3	1.9	
	S20	Does your child usually drink sugary drinks?	Never	21	13.1	
			Sometime	108	67.5	
			Everyday	31	19.4	
	S21	What does your child eat for lunch at school?	Pasta/rice	3	1.9	
			Meat/fish	53	33.1	
			Eggs	20	12.5	
			Fruit/vegetables	29	18.1	
			Pizza, chips, fast food	28	17.5	
			Sandwich (ham & cheese)	12	7.5	
			Sweets/cakes	8	5.0	
			Others	7	4.4	
	S22	What does your child drink during lunch?	Water	142	88.8	
			Fruit juice	18	11.2	
	S23	Does your child drink sugary beverages before going to bed at night?	No, never	119	74.4	
			Sometime	33	20.6	
			Yes, always	8	5.0	
	S24	If so, does he/she brush his/her teeth after?	No, never	119	74.4	
			Sometime	28	17.5	
			Yes, always	13	8.1	
	S25 *	Do you have control over your child’s eating habits? * Chi-square 101.712 (*p* = 0.000)	No	3	1.9	
			Sometime	50	31.2	
			Yes	107	66.9	
Oral hygiene habits	S26	How many times a day does your child brush his/her teeth?	1time	50	31.2	91.350 (*p* = 0.000)
			2time	104	65.0	
			3 time	6	3.8	
	S27	What type of brush does your child use?	Manual	130	81.2	
			Electric	30	18.8	
	S28	Does your child use any other oral hygiene devices, besides tooth brush ?	No	145	90.6	
			Yes	15	9.4	
	S29	Presence of caries	Yes	25	15.6	
			No	135	84.4	
	S30	Type of decayed tooth	Incisors and canines	3	1.9	
			Deciduous molars	6	3.8	
			None	151	94.4	
Clinical data on obturated teeth	S31	Number of restorations	None	151	94.4	449.625 (*p* = 0.000)
			1-3 restorations	8	5.0	
			4 restorations or more	1	0.6	
	S32	Type of filled tooth	Incisors or canines	11	6.9	
			Deciduous molars	1	0.6	
			None	148	92.5	
Clinical data on malocclusion	S33	Presence of malocclusion?	No	111	69.4	490.100 (*p* = 0.000)
			Yes	49	30.6	
	S34	Type of malocclusion	Open-bite	23	14.4	
			Deep-bite	6	3.8	
			Unilateral crossbite/bilateral crossbite	12	7.5	
			Anterior crossbite/functional third class	2	1.2	
			Crowding	2	1.2	
			Functional second class	4	2.5	
			None	111	69.4	

**Table 3:** Multivariate logistic regression.

Model	Coefficient B	R	Adjusted R Square	Beta	*p*-Value
Predictors: (constant), dental visits	0.281	0.304	0.087	0.304	0.000
Predictors: (constant), clinical data on obturated teeth	−0.287	0.234	0.055	−0.234	0.003

**Table 4:** Univariate logistic regression.

Model	Coefficient B	R	Adjusted R Square	Beta	*p*-Value
Predictors: (constant), how many meals does your child eat per day?	0.151	0.193	0.037	0.193	0.015
